# Nicotinamide Mononucleotide Potentiates Resistance to Biotrophic Invasion of Fungal Pathogens in Barley

**DOI:** 10.3390/ijms22052696

**Published:** 2021-03-07

**Authors:** Kana Ueda, Yuichi Nakajima, Hiroshi Inoue, Kappei Kobayashi, Takumi Nishiuchi, Makoto Kimura, Takashi Yaeno

**Affiliations:** 1 Department of Agriculture, Ehime University, Tarumi, Matsuyama, Ehime 790-8566, Japan; e611008x@mails.cc.ehime-u.ac.jp (K.U.); g651001k@mails.cc.ehime-u.ac.jp (H.I.); kobayashi.kappei.mk@ehime-u.ac.jp (K.K.); 2 Division of Molecular and Cellular Biology, Graduate School of Bioagricultural Sciences, Nagoya University, Furo-cho, Chikusa, Nagoya, Aichi 464-8601, Japan; ynakajima.gm@gmail.com (Y.N.); mkimura@agr.nagoya-u.ac.jp (M.K.); 3 Institution for Gene Research, Advanced Science Research Centre, Kanazawa University, 13-1 Takara-machi, Kanazawa, Ishikawa 920-0934, Japan; tnish9@staff.kanazawa-u.ac.jp

**Keywords:** nicotinamide mononucleotide, *Fusarium graminearum*, *Blumeria graminis* f. sp. *hordei*

## Abstract

Nicotinamide mononucleotide (NMN), a precursor of nicotinamide adenine dinucleotide (NAD), induces disease resistance to the Fusarium head blight fungus *Fusarium graminearum* in *Arabidopsis* and barley, but it is unknown at which stage of the infection it acts. Since the rate of haustorial formation of an obligate biotrophic barley powdery mildew fungus *Blumeria graminis* f. sp. *hordei* (*Bgh*) was significantly reduced in NMN-treated coleoptile epidermal cells, the possibility that NMN induces resistance to the biotrophic stage of *F. graminearum* was investigated. The results show that NMN treatment caused the wandering of hyphal growth and suppressed the formation of appressoria-like structures. Furthermore, we developed an experimental system to monitor the early stage of infection in real-time and analyzed the infection behavior. We observed that the hyphae elongated windingly by NMN treatment. These results suggest that NMN potentiates resistance to the biotrophic invasion of *F. graminearum* as well as *Bgh*.

## 1. Introduction

*Fusarium graminearum* is the causal agent of Fusarium head blight (FHB) in barley and wheat. Fungal toxins such as nivalenol (NIV), deoxynivalenol (DON), and their derivatives produced by *Fusarium* species are toxic to humans, animals, and plants, resulting in a loss of yield and reduced quality of grains [1]. Several genes involved in resistance to FHB have been found, but their application to breeding will take a long time. Although it is possible to control barley and wheat by treating them with fungicides at the flowering stage, fungicide residues are undesirable for humans and animals. Furthermore, the abuse of fungicides leads to the generation of fungicide-resistant *Fusarium* strains, which is not suitable for sustainable agriculture [2]. Plant activators are compounds that protect crops from disease by activating the plant’s immune system. Benzo (1,2,3) thiadiazole-7-carbothionic acid S-methyl ester, a plant activator that acts like salicylic acid (SA), does not have antimicrobial activity by itself but confers disease resistance against the hemibiotrophic bacterial pathogen *Pseudomonas syringae* pv. *tomato* DC3000 and *F. graminearum* by inducing the expression of genes related to systemic acquired resistance in plants [3,4,5]. Other plant activators, such as nicotinamide adenine dinucleotide (NAD) and nicotinamide adenine dinucleotide phosphate (NADP), have been reported to induce the expression of disease resistance-related (*PR*) genes via the calcium-dependent signaling, and enhance the resistance to *P. syringae* pv. *maculicola* ES4326 through a lectin receptor kinase/Brassinosteroid insensitive1-Associated Kinase1 complex [6,7]. Inoculation of barley with an obligate biotrophic fungal pathogen *Blumeria graminis* f. sp. *hordei* (*Bgh*) also significantly increases the amount of NAD, suggesting that NAD biosynthesis is involved in disease resistance [8]. Nicotinamide mononucleotide (NMN), a precursor of NAD biosynthesis that exerts anti-aging effects in animals via the sirtuin gene encoding a NAD-dependent protein deacetylase, was found to improve disease resistance to *F. graminearum* in *Arabidopsis* [9,10]. Furthermore, exogenous NMN application significantly reduced disease development and DON production by *F. graminearum* in barley, suggesting its potential use as a plant activator. However, it is not yet known how NMN affects the infection process of *F. graminearum*.

In the field, airborne spores of *F. graminearum* germinate on flowering spikelets of barley or wheat and enter through natural openings such as crevices at the base of lemma, palea, or stomata on the epidermis [11]. However, hyphal growth on the epidermis before entry through crevices in lemma and palea in barley and direct entry into the epidermis of lemma and palea in wheat were also observed [12,13]. Recently, it has also been shown that *F. graminearum* can enter from trichomes of several hosts [14,15]. Subsequently, the infection process was divided into three stages by further detailed microscopic observations [16]. In the early colonization stage (Stage I), the germination and hyphal elongation occur within 6–12 h post-inoculation (hpi). Short infection hyphae for direct penetration are observed. Homogeneous hyphae are formed on caryopses until 1–2 days post-inoculation (dpi), on paleas until 4–5 dpi, and on lemmas and glumes until 6–7 dpi. After each time point, the hyphal morphology becomes heterogeneous. During the main stage of infection (Stage II), the runner hyphae branch at a high frequency, forming foot structures, lobate appressoria, and infection cushions. These structures are observed at 3 dpi on caryopses, 5 dpi on paleas, and 7 dpi on glumes. DON is thought to be synthesized at the time of infection cushion formation. Brown necrosis and chlorosis develop during this stage. In the final stage (Stage III), the entire spikelets are necrotic and aerial hyphae and sporodochia are formed. However, observation with an optical microscope is limited by the fluorescence of the chlorenchyma of the spikelets, which obscures the morphology of the multilayered hyphae. Therefore, further detailed analysis of Stage I and Stage II was performed by confocal fluorescence microscopy using an *F. graminearum* strain expressing GFP constitutively and the epidermis of wheat coleoptiles, which are more easily observed due to its single-cell layer [17]. The coleoptile epidermal cells have been used as a suitable material to observe the morphogenesis of *Bgh*, which occurs relatively synchronously on time [18]. For example, the primary germ tube emerges within 2 h after the conidium comes into contact with the host’s epidermal cell, and the second germ tube, the appressorial germ tube (AGT), forms 8–10 h later. About 2 h after the formation of AGT, the tip of the AGT hooks to form an appressorium for penetration. In the study of *Bgh*, which only infects epidermal cells, the infection process is very easy to observe because the epidermal cells of the coleoptile are a single-cell layer. Although the morphology of the hyphae of *F. graminearum* is more heterogeneous than that of *Bgh*, the coleoptile epidermal cells are also suitable for observing the microscopic structures involved in the infection process, since the fungus penetrates epidermal cells. In the early stages of infection (5–8 hpi), conidia of *F. graminearum* germinate and elongate the runner hyphae on the extracellular surface or the intercellular spaces of the coleoptiles. The runner hyphae do not immediately penetrate the host cells but form the appressoria-like structures (also called foot structures). Infectious hyphae penetrating the epidermal cells are observed by 16 hpi. After penetration, the hyphae continue to grow inside the cells, branching out and forming bulbous structures. After about 36 hpi, the hyphae form more invasive hyphae and spread to neighboring cells. Since the host cells are still alive at this time, the lifestyle of *F. graminearum* is considered to be biotrophic. As the infection stage progresses further, it shifts to Stage II, which is necrotrophic [17].

In this study, based on the finding that NMN pretreatment effectively suppress the production of reactive oxygen species (ROS) for necrotrophic growth of *F. graminearum* in *Arabidopsis* [9], we investigated whether NMN enhances the defense response against biotrophic invasion in the stage before the necrotrophic stage. To effectively treat cells with NMN and facilitate morphological observation of *F. graminearum*, we established a real-time monitoring system for infection behavior of the GFP-expressing strain using a single layer of barley coleoptile epidermal cells. In addition to inhibiting the penetration of *Bgh*, NMN was found to be effective in inhibiting the formation of appressoria-like structures and inducing wandering of the hyphal growth in *F. graminearum*. Taken together, NMN may function as a plant activator that potentiates resistance to the biotrophic stage of fungal pathogens.

## 2. Results and Discussion

### 2.1. NMN Induces Penetration Resistance to Bgh

**Figure 1:**
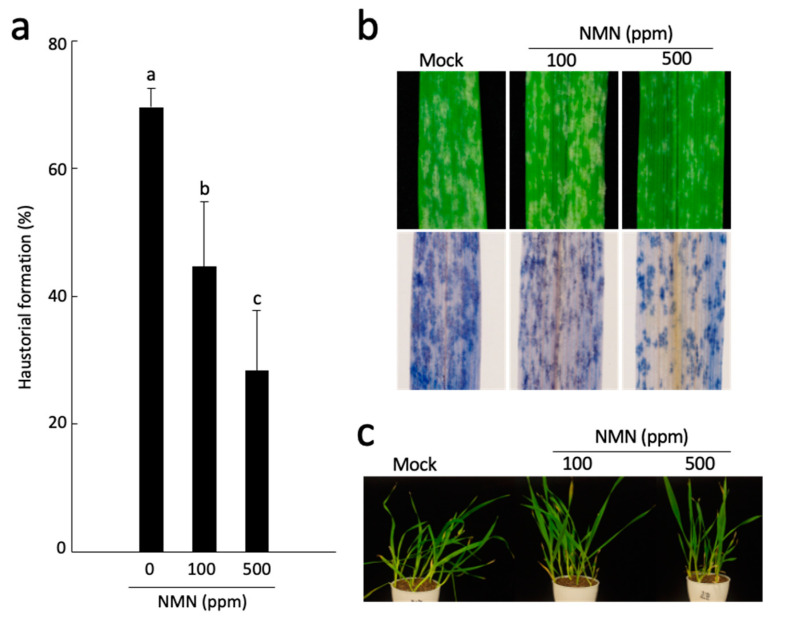
NMN induces penetration resistance to the barley powdery mildew fungus *Blumeria graminis* f. sp. *hordei* (*Bgh*). (**a**) The penetration rate of *Bgh* in NMN-treated epidermal cells of barley coleoptile was calculated by counting the number of conidia developing a haustorium per the number of interactions between an epidermal cell and a conidium attacking the cell with an appressorium. Bars indicate the standard deviation (*n* = 7). Different letters indicate significant differences (*p* < 0.05; Tukey’s test). (**b**) NMN-treated leaves inoculated with *Bgh* were photographed after 4 days and stained with trypan blue solution. (**c**) NMN treatment did not cause growth inhibition even at 500 ppm.

NMN, a precursor of NAD, enhances resistance to *F. graminearum* [9], and NAD levels increase upon infection by *Bgh* [8], suggesting that NMN is also involved in resistance to *Bgh*. Therefore, we first investigated the effect of NMN on the resistance using epidermal single-cell layers of coleoptiles, which are easy to treat with chemicals and observe during the infection process. *Bgh*, an obligate biotrophic fungus, penetrates the host epidermal cells and forms haustorium, which must absorb nutrients to survive. Therefore, the haustorial formation is a criterion to evaluate whether the infection is successful or not. The coleoptile epidermal cells prepared as a single-cell layer are in direct contact with chemicals because there is no cuticle on their lower surface. Inoculation of *Bgh* conidia after 12 h of NMN pretreatment resulted in a significant decrease in the rate of haustorial formation in a concentration-dependent manner (Figure 1a). Additionally, when NMN was administered by absorption from the roots, the effect of NMN was observed even though the conidia were inoculated at a high density (Figure 1b). Since the infection rate of *Bgh* decreases when the host is not healthy, we investigated the possibility that NMN treatment might affect plant growth and found that NMN did not inhibit growth even at 500 ppm, where the effect was pronounced (Figure 1c). These results suggest that NMN has an inhibitory effect on biotrophic invasion by *Bgh*.

### 2.2. NMN Interferes with the Biotrophic Process of F. graminearum Infection

**Figure 2:**
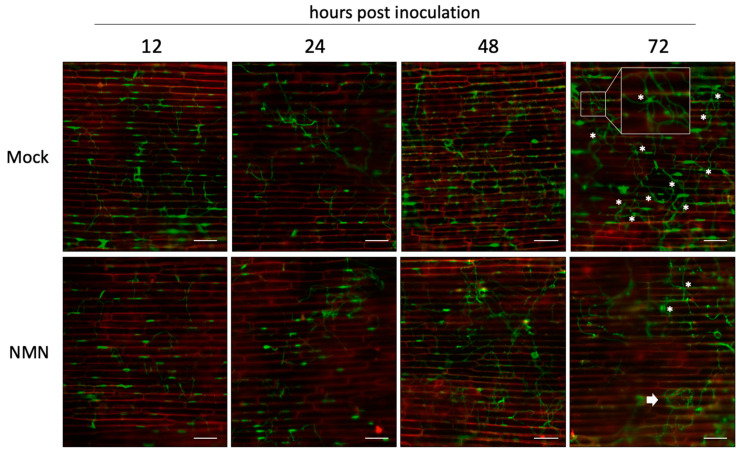
Infection behavior of *Fusarium graminearum* on coleoptile epidermal cells treated with NMN. The coleoptile epidermal cells were treated with 500 ppm NMN and inoculated with conidia of GFP-expressing *F. graminearum*. After inoculation, plasma membranes of epidermal cells were stained with FM4-64. The asterisks indicate hyphal entanglements. The square enclosure shows the magnified hyphal entanglement. The arrow indicates typical wandering hyphae with many turning points. Scale bars = 100 µm.

To analyze the infection behavior of *F. graminearum* on coleoptile epidermal cells, we used a strain that constitutively expresses GFP. The coleoptile epidermal cells were inoculated 1 h after treatment with 500 ppm NMN, and sequential morphological changes were observed. The plasma membranes were stained with FM4-64 to facilitate observation of the host epidermal cell shape. A couple of hours after inoculation, conidia germinated, and hyphae elongated vigorously. After that, however, hyphae tended to grow slightly weaker on NMN-treated coleoptile epidermal cells (Figure 2).

**Figure 3:**
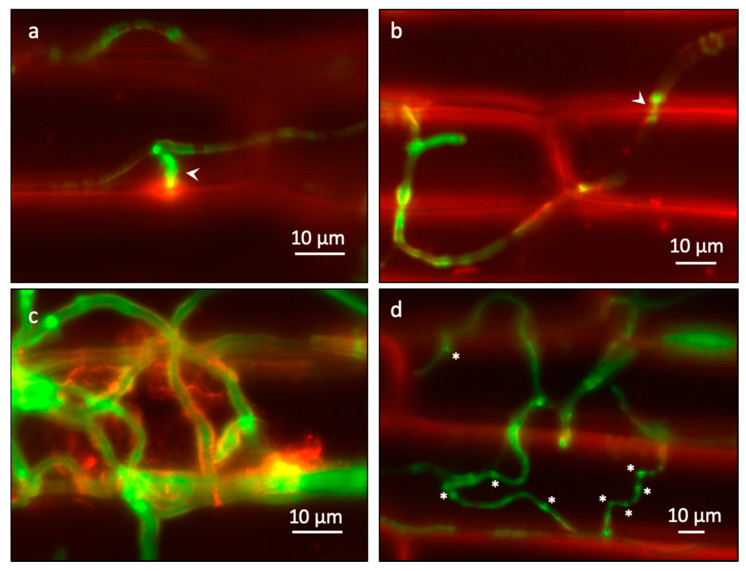
Hyphal structures of *Fusarium graminearum* on coleoptile epidermal cells. (**a**) The appressoria-like structure of *F. graminearum* on the mock-treated cells 12 h post inoculation (hpi). The arrowhead indicates an appressorium-like structure. (**b**) The invaded hypha penetrates the neighboring cell of the mock-treated coleoptile 24 hpi. The arrowhead indicates a constricted invasive hyphal peg. (**c**) The invasive hyphae surrounded by the host cell membranes of the mock-treated coleoptile 24 hpi. (**d**) The wandering hyphae on the coleoptile epidermal cells treated with 500 ppm NMN 12 hpi. The asterisks indicate the turning points with slight strong fluorescence.

On the mock-treated cells, the appressoria-like structures were observed at 12 hpi (Figure 3a). Twenty-four hours after inoculation, biotrophic invasion processes, such as invasive hyphae pegs, where hyphae invaded a cell and spread to neighboring cells, and invasive hyphae surrounded by the host cell membrane, were observed (Figure 3b,c). However, wandering hyphae were frequently observed on NMN-treated epidermal cells of coleoptiles even at 12 hpi, as if they were hesitant to penetrate the host cells (Figure 3d). In such hyphae, slightly stronger fluorescence tended to be observed at points where the direction of elongation changed and where appressoria-like structures showed signs of forming but had not yet visibly formed. The slightly strong fluorescence at the turning points is distinguishable from that in the mature appressoria-like structures (Figure 3d, white asterisks, and 3a, respectively).

**Figure 4:**
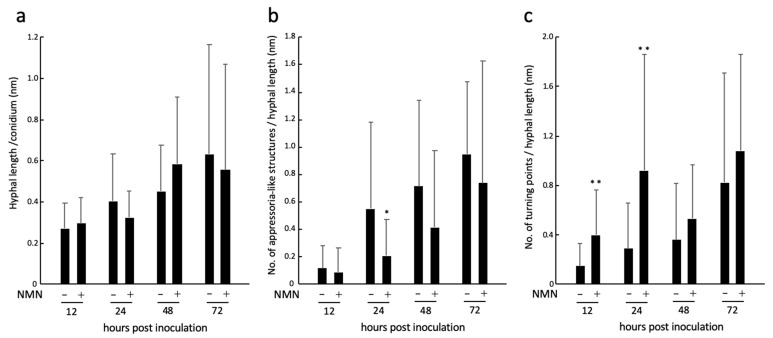
Morphology of *Fusarium graminearum* on coleoptile epidermal cells treated with 500 ppm NMN. (**a**) The hyphal length of *F. graminearum.* (**b**) The appressoria-like structures per hyphal length. The asterisk indicates a significant difference between control and NMN treatments (*p* < 0.05; Student’s t-test). (**c**) The turning points per hyphal length. The double-asterisk indicates a significant difference between control and NMN treatments (*p* < 0.01; Student’s t-test). Bars indicate the standard deviation (*n* = 24).

Hence, we investigated in detail how NMN treatment affects the morphology of *F. graminearum*. When the length of hyphae per conidium at each time was compared, there was no significant difference between the mock and NMN treatments (Figure 4a). Next, we examined how many appressoria-like structures were formed on the hyphae. The results show that the number of appressoria-like structures was significantly reduced by NMN treatment at 24 hpi (Figure 4b). Appressoria-like structures are also observed in wheat as early infection structures that adhere tightly to the surface of coleoptile epidermal cells [17]. Similarly, the structures are also formed during penetration of neighboring epicarp cells from hyphae within the invaded cells [19]. *F. graminearum* can penetrate through the tip of a straight hypha, so a distinctly formed appressoria-like structure may be dispensable but could help increase the chance of penetration by making the small hyphal branches. The rice blast fungus *Magnaporthe oryzae* increases the turgor pressure inside the appressorium to 8.0 MPa and breaks through the leaf cuticle surface by physical rupture [20]. On the other hand, the turgor pressure inside the appressorium is not so high in *Bgh*, and melanin is not accumulated to maintain the turgor pressure. Therefore, it would not be only physical forces that penetrate the cell wall [21]. *Bgh* may secrete proteins necessary for penetration from the tip of the appressorium [18]. Likewise, *F. graminearum* probably secretes proteins such as cell-wall-degrading enzymes from the tips of the appressoria-like structures in order to penetrate host cells. The co-localization of the tip of the appressoria-like structure with the host cell membrane stained with FM4-64 suggests that it interacts with the host (Figure 3a). The number of turning points was significantly higher on NMN-treated coleoptile epidermal cells at 12 and 24 hpi (Figure 4c). In the process of elongation, hyphae encounter host responses induced by NMN, which are unfavorable to *F. graminearum*, possibly making it difficult for direct penetration or subsequent formation of mature appressoria-like structures at 24 hpi (Figure 4b). As a result, hyphae may exhibit a wandering morphology (Figure 2 and Figure 3d). After 72 h of inoculation, each hypha could grow on the NMN-treated coleoptile epidermal cells as in the control, but the degree of hyphal entanglement was low (Figure 2). In Stage II, hyphae elongate in all directions on the surface of the epidermis and become entangled with each other to form lobate appressoria and consequent infection cushions [16]. The NMN-mediated suppression of disease in spikelets [9] suggests that NMN could affect these fungal growth phases, though this remains to be studied. It is not feasible to quantitate the degree of entanglement. The lifespan of single-layered coleoptile epidermal cells is not long enough to observe the infection cushion formation. Qiu et al. (2019) also did not observe the formation of infection cushions in wheat coleoptile epidermal cells. Therefore, the development of a new experimental system using lemma and palea is necessary to evaluate NMN effects on hyphal entanglement and infection cushion formation after Stage II.

### 2.3. Real-Time Imaging of Infection Behavior of F. graminearum

**Figure 5:**
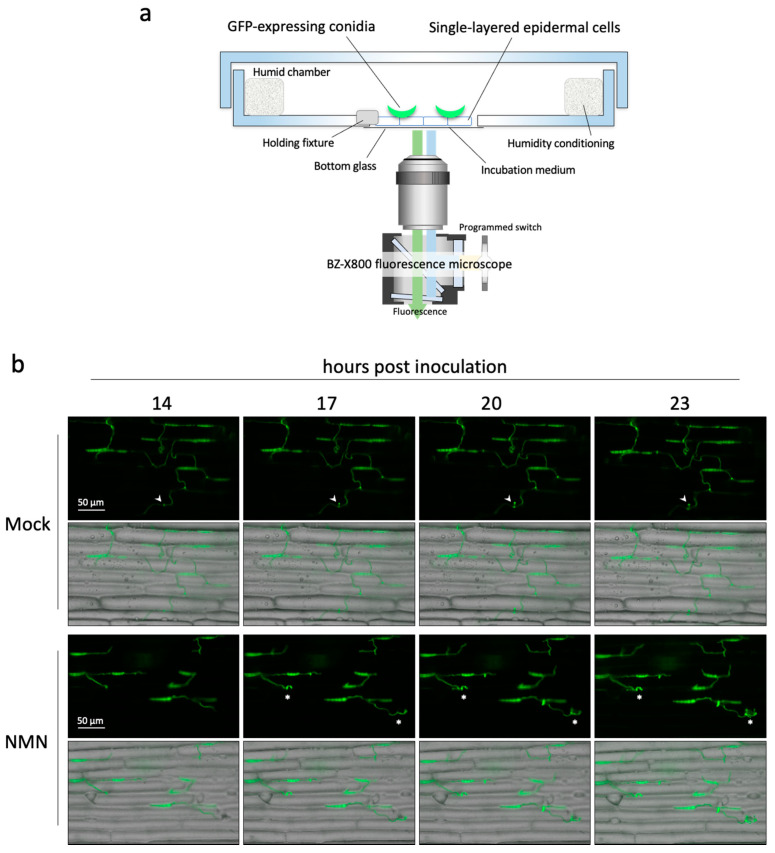
Real-time imaging of infection behavior of *Fusarium graminearum* on coleoptile epidermal cells treated with 500 ppm NMN. (**a**) Real-time monitoring system for infection behavior of *F. graminearum*. (**b**) Three hours after inoculation and thereafter, the fluorescence was automatically photographed every 30 min. The real-time imaging was supplemented as video files (Videos S1–S4). The arrowheads indicate an appressorium-like structure. The asterisks indicate winding hyphae.

We found that NMN treatment affected the elongation of hyphae and the formation of appressoria-like structures. However, since these results were obtained at different time points, it is necessary to observe the infection behavior continuously to clarify how NMN treatment affects it. Therefore, we developed an inoculation system for real-time imaging of infection behavior (Figure 5a). Since the lower surface of the single-layered coleoptile epidermal cells has no cuticle, they die easily when they dry out. Any slight movement of the position of the cell layer due to drying causes them to lose focus. Additionally, high humidity is necessary for the infection of *F. graminearum*. For these reasons, a glass-bottom dish was used to prevent evaporation of the NMN solution and to maintain high humidity while observing. Moistened papers were placed around the specimen to make a humid chamber throughout the real-time imaging. Three hours after inoculation, the coleoptile epidermal cell layer was set in the darkroom stage of the fluorescence microscope and automatically photographed every 30 min. In the mock treatment, the hyphae grew relatively straight and gradually formed appressoria-like structures. On the other hand, in the NMN treatment, the hyphae grew but elongated windingly (Figure 5b, Videos S1–S4). These results suggest that NMN treatment induces a state in which the hyphae of *F. graminearum* are less likely to branch and form appressoria-like structures.

NMN treatment activates SA-dependent signaling, which positively regulates resistance to *F. graminearum* in *Arabidopsis* [9]. SA was also reported to be a signaling molecule that positively regulates resistance to the fungus in barley [22]. The silencing of *HvICS*, which is thought to encode a key enzyme for SA biosynthesis in barley, compromises the resistance to *F. graminearum* as well as *Bgh*, indicating that SA contributes to resistance to the biotrophic invasion of these fungi. The fact that *HvICS* overexpression suppresses the growth of *F. graminearum* in the early stage of infection (24 and 48 hpi) but not in the late stage (72 hpi) suggests that SA acts specifically in the biotrophic stage. Furthermore, the suppression of ROS accumulation by *HvICS* overexpression is consistent with the suppression of ROS accumulation in the necrotrophic stage by NMN treatment [9,22]. Taken together, NMN may potentiate resistance to the biotrophic invasion of *F. graminearum* in coleoptile epidermal cells by activating SA-dependent signaling. In the future, it will be necessary to clarify which genes are induced by NMN and contribute to the resistance.

## 3. Materials and Methods

### 3.1. Plant and Fungal Materials

Seedlings of barley (*Hordeum vulgare* cv. Kobinkatagi) were grown in growth chambers (NK system LH-200-RD, Nippon Medical & Chemical Instruments Co., Ltd., Osaka, Japan) at 20 °C under the continuous light condition as described in Kawamoto et al. [23]. Coleoptiles were excised from seedlings and single-cell epidermal layers of partially dissected coleoptiles were prepared as described previously [24]. Powdery mildew fungus *Bgh* RACE1 isolate was cultured on the barley leaves in culture chambers (NK system LH-200-RD, Nippon Medical & Chemical Instruments Co., Ltd., Osaka, Japan) at 20 °C as described previously [25]. *F. graminearum* strain JCM 9873 was used in this study. The strain with constitutive expression of *GFP* by *TEF1α* promoter was generated as described by Nakajima et al. [26]. A *GFP* fragment was amplified from pP*_TEF1__α_*_*Tri6*::*EGFP* by polymerase chain reaction with primers GFP-HindIII_S (5′-TCTAAGCTTATGGTGAGCAAGGGCGAGGA-3′) and GFP-SpeI_AS (5′-TCTACTAGTTCACTTGTACAGCTCGTCCA-3′), and the amplified fragment was inserted into HindIII/SpeI sites of pAnTef-hph. The resulting plasmid pP*_TEF1__α_*_*EGFP* was linearized by XhoI digestion and introduced to *F. graminearum* by the protoplast-PEG method.

### 3.2. Inoculation of Bgh

The coleoptiles were incubated on a Kimwipe paper moistened with a 1 mM CaCl_2_ solution containing NMN 12 h before inoculation. The coleoptiles were then inoculated with *Bgh* conidia using a brush and incubated at 20 °C in dark conditions for about 30 h. The penetration rate was calculated by counting the number of conidia developing a haustorium per the number of interactions between an epidermal cell and a conidium attacking the cell with an appressorium as described in Sugai et al. [18]. When studying the effect of NMN on leaves, seeds were grown on filter paper containing NMN solution for 12 days, transferred to vermiculite, and grown for 20 days. Four days after inoculation with *Bgh* at a concentration of more than 400 conidia per cm^2^, disease symptoms were photographed. Trypan blue staining was performed as described in Yaeno et al. [27].

### 3.3. Inoculation of F. graminearum

A portion of conidial suspension stored as a glycerol stock at −80 °C (10^7^ spores/mL in 30% glycerol) was incubated in CMC medium (1.5% (*w/v*) carboxymethyl cellulose sodium salt, 0.1% (*w/v*) NH_4_NO_3_, 0.1% (*w/v*) KH_2_PO_4_, 0.05% (*w/v*) MgSO_4_ heptahydrate, and 0.1% (*w/v*) yeast extract) with gentle shaking at 25 °C for 2 days, and the fresh conidia were obtained as an inoculum as described in Maeda et al. [28]. The conidia were filtrated through a cell strainer, suspended in sterile distilled water, washed centrifugally, and then resuspended in sterile distilled water to a concentration of 5 × 10^5^ conidia/mL. Five microliters of the inoculum were dropped onto the prepared epidermal cell layer of coleoptiles and excess liquid was removed with Kimwipe paper. After inoculation, coleoptiles were incubated at 25 °C in dark conditions.

### 3.4. Real-Time Monitoring of Infection Behavior of F. graminearum

Inoculated coleoptile was placed onto a glass-bottom culture dish (35 mm in diameter). To avoid drying out, 1 mM CaCl_2_ was supplied under the coleoptile. To maintain high humidity inside, small pieces of moistened Kimwipe paper were placed around the coleoptile and the lid of the dish was sealed with Parafilm (Figure 5a). Images of infection behavior were taken under a BZ-X800 all-in-one fluorescence microscope (Keyence, Osaka, Japan). Real-time monitoring was automatically performed by capturing fluorescence images of 17 Z-stacks every 30 min for 48 h using the long-term time-lapse modules (BZ-H4XD and BZ-H4XT, Keyence). A time-lapse movie was generated with BZ-X800 Analyzer (BZ-H4A, Keyence). Green fluorescence of *F. graminearum* expressing GFP was detected using the GFP filter (OP-87763, Keyence), and red fluorescence of FM4-64 staining the plasma membrane of epidermal cells was detected using the TRITC filter (OP-87764, Keyence).

## Data Availability

The data presented in this study are available in article and Supplementary Material.

## Supplementary Materials

The following are available online at https://www.mdpi.com/1422-0067/22/5/2696/s1, Video S1: Fluorescence image of GFP-expressing *F. graminearum* in mock-treated coleoptile epidermal cells, Video S2: Fluorescence image of GFP-expressing *F. graminearum* in mock-treated coleoptile epidermal cells merged with the brightfield image, Video S3: Fluorescence image of GFP-expressing *F. graminearum* in NMN-treated coleoptile epidermal cells Video S4: Fluorescence image of GFP-expressing *F. graminearum* in NMN-treated coleoptile epidermal cells merged with the brightfield image.
